# Pancreatic serous cystoadenoma (CSA) showing increased tracer uptake at 68-GaDOTA-peptide Positron Emission Tomography (68Ga-DOTA-peptide PET-CT): a case report

**DOI:** 10.1186/s12893-020-01004-2

**Published:** 2020-12-14

**Authors:** Gennaro Nappo, Niccola Funel, Simone Giudici, Paola Spaggiari, Giovanni Capretti, Silvia Carrara, Giovanna Pepe, Alessandro Zerbi

**Affiliations:** 1 Pancreatic Surgery Unit, Humanitas Clinical and Research Center-IRCCS Rozzano, Via Alessandro Manzoni, 56, 20089 Rozzano, Milan, Italy; 2 Department of Translational Research and New Technologies in Medicine and Surgery, University of Pisa, Pisa, Italy; 3 Pathology Department, Humanitas Clinical and Research Center-IRCCS Rozzano, Milan, Italy; 4 Endoscopic Unit, Humanitas Clinical and Research Center-IRCCS Rozzano, Milan, Italy; 5 Nuclear Medicine Department, Humanitas Clinical and Research Center-IRCCS Rozzano, Milan, Italy; 6 Humanitas University, Rozzano, Milan Italy

**Keywords:** Pancreatic serous cystoadenoma, Ga68-DOTA-peptide PET-CT, Pancreatic cystic lesions, Pancreatic neuroendocrine neoplasms, Case report

## Abstract

**Background:**

Serous cysto-adenoma (SCA) is a rare benign neoplasm of the pancreas. SCA can mimic other pancreatic lesions, such as neuroendocrine tumours. 68Gallium-DOTA-peptide Positron Emission Tomography (PET) is able to image in vivo the over-expression of the somatostatin receptors, playing an important role for the identification of neuroendocrine neoplasms.

**Case presentation:**

We reported a case of 63-year-old man*,* with a solid lesion of 7 cm of diameter of the body–tail of the pancreas. Two fine-needle-aspirations (FNA) were inconclusive. A 68Ga-DOTA-peptide PET-CT revealed a pathological uptake of the pancreatic lesion. The diagnosis of a pancreatic neuroendocrine neoplasm was established and a laparoscopic distal splenopancreatectomy and cholecystectomy was performed. Final histopathological report revealed the presence of a micro-cystic SCA.

**Conclusions:**

The current case firstly reports a pancreatic SCA showing increased radiopharmaceutical uptake at 68Ga-DOTA-peptide PET-CT images. This unexpected finding should be taken into account during the diagnostic algorithm of a pancreatic lesion, in order to minimize the risk of misdiagnosis and overtreatment of SCA.

## Background

Serous cysto-adenoma (SCA) is a rare benign neoplasm of the pancreas [1]. SCA can mimic other pancreatic lesions: about 10% of pancreatic neuroendocrine tumours (pNET) appears cystic on imaging and can be misdiagnosed with SCA [2]. 68Gallium-DOTA-peptide Positron Emission Tomography (PET) is able to image in vivo the over-expression of the somatostatin receptors, playing an important role for the identification of neuroendocrine neoplasms [3].

The following case-report firstly describes a pancreatic SCA showing uptake to 68-Gallium-DOTA-peptide. This case confirms the diagnostic challenge offered by pancreatic cystic neoplasms and encourages future investigations on the usefulness of 68Ga-DOTA-peptide in discriminating pancreatic SCA from pancreatic NET.

The following case report was written following the CARE Guidelines [4].

## Case presentation

**Fig. 1:**
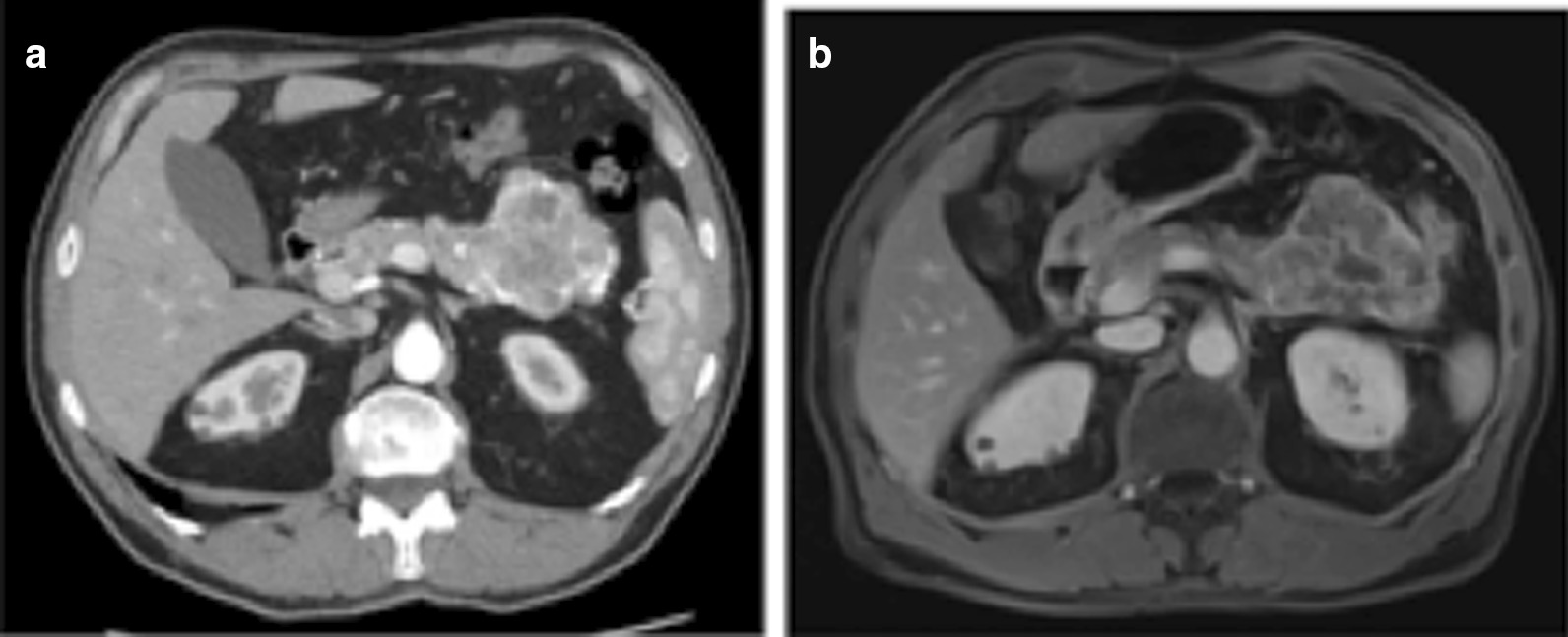
**a** CT-scan showing a lesion of 7 cm of diameter in the body and tail of the pancreas; **b** IRM confirming an expansive solid lesion, with multiple septa inside, strictly adherent to the jejunum

**Fig. 2.**
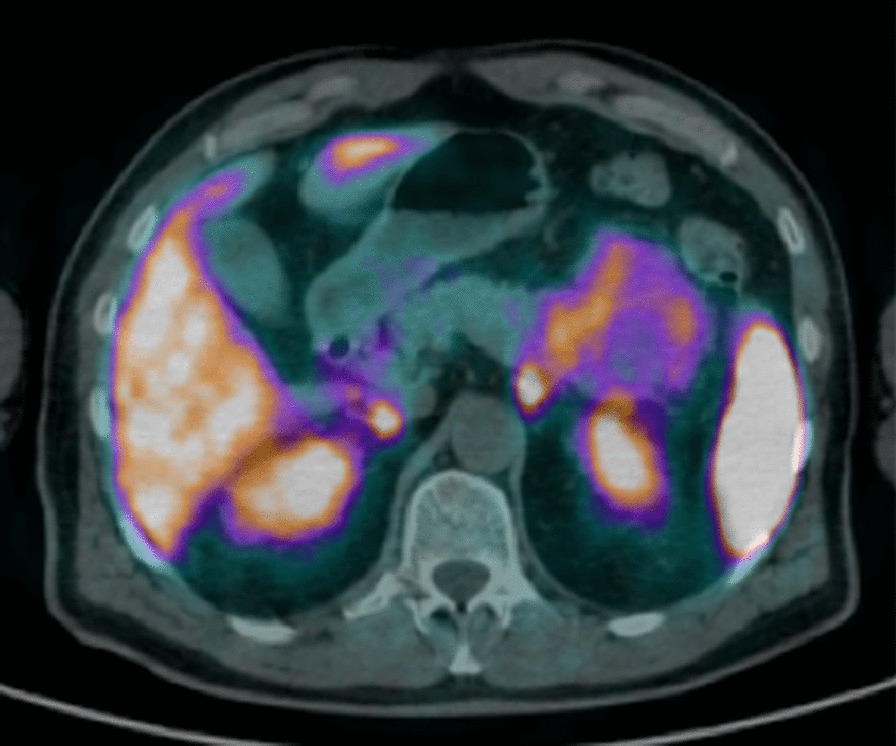
68Ga-DOTA-peptide (namely 68GaDOTATOC) PET-CT scan revealing a pathological uptake of the radiopharmaceutical in the pancreatic lesion

**Fig. 3:**
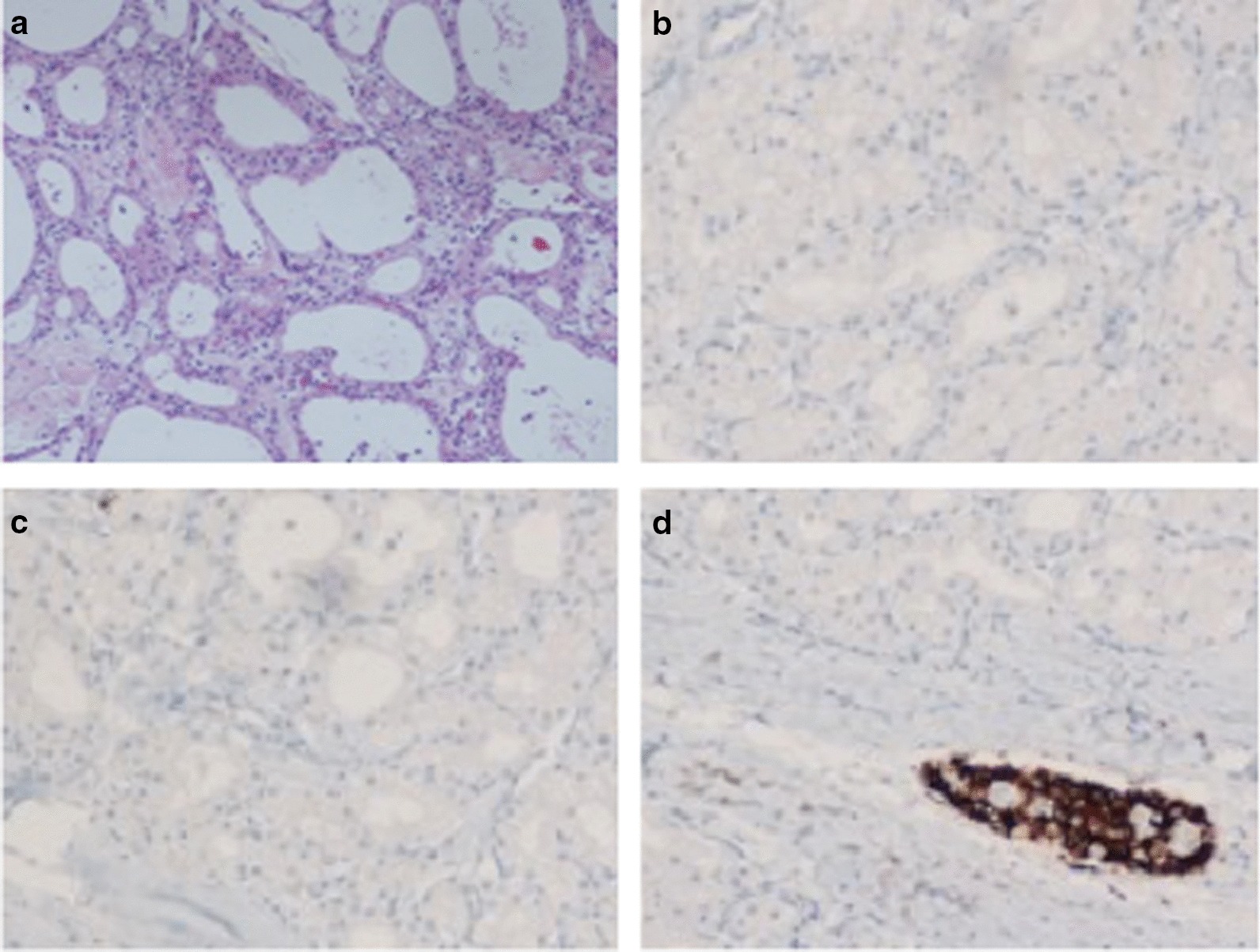
Histology of the specimen: **a** the cysts were lined by a single layer of uniform, cuboidal, epithelial cells with clear cytoplasm (due to abundant glycogen) and central oval hyperchromatic nuclei with inconspicuous nucleoli; the central scar and radiating septa was composed of hyalinised collagen. Hematoxylin and Eosin staining (magnification 10×); **b**–**d** Immuno-histochemical analysis for Synaptophisin (**b**), Vimentin (**c**) and Chromogranin (**d**), in which, only Langerhans’ islet (Red arrow), and not the cystic lesion (Blue arrows), showed positivity for chromogranin A

A 63-year-old man*,* without relevant past medical history, was referred to undergo an abdominal computed tomography scan (CT), due to a diffuse mild abdominal pain. The patient describes that the pain hadn’t any specific feature, not aggravating or alleviating from some specific factors, it appeared one time each week, it wasn’t invalidating and it disappeared without adoption of any medications. No fever, vomiting, diarrhea or weight loss were present. CT images showed a lesion sized 7 cm of diameter of the body–tail of the pancreas (Fig. 1a). An Endoscopic Ultrasound (EUS) showed a vascularized hypoechoic lesion with exophytic growth in the body of the pancreas. A fine needle aspiration (FNA) was performed, showing the presence of epithelial cells, without atypia (category II according the Papanicolaou Society of Cytopathology Guidelines) [5]. One month after, a new EUS with FNA was repeated, but the results was inconclusive (category I according the Papanicolaou Society of Cytopathology Guidelines) [5]. In both endoscopic examinations, a dosage of intra-cystic glucose, CA 19.9 and CEA was not performed. A magnetic resonance (MRI) was performed, confirming the expansive solid lesion, with multiple septa inside, strictly adherent to the jejunum (Fig. 1b). Laboratory tests were normal, as well as Ca 19.9 value. After these investigations, we had two diagnostic theories: (1) a pancreatic SCA, considering the presence multiple septa delimitating small cystic spaces, even if the typical central scar was missing; (2) a pancreatic NET, due to the presence of a solid lesion highly vascularized. In order to discriminate these two disease, 2 months after, a 68Ga-DOTA_peptide PET-CT was performed, revealing a pathological uptake of the pancreatic lesion (SUV max 9.6; mean 7.1) (Fig. 2). A diagnosis of pancreatic neuroendocrine tumour was established, with consequently indication to surgery [6]. A distal splenopancreatectomy and cholecystectomy with laparoscopic approach was performed: intraoperatively, we confirmed the presence of a large solid mass, hyper-vascularized, strictly adherent to mesocolon. The lesion was extremely attached to splenic vessel and near to the spleen: for these reasons, was not possible to performed a distal pancreatectomy spleen preserving. Post-operative course was uneventful and the patient was discharged on post-operative day (POD) [7]. Final histopathological report was diagnostic for microcystic CSA: the cysts were lined by a single layer of uniform, cuboidal, epithelial cells with clear cytoplasm (due to abundant glycogen, demonstrated at histochemical analysis by the positivity for Periodic Acid-Schiff (PAS) and negativity for PAS-diastase) and central oval hyperchromatic nuclei with inconspicuous nucleoli; the central scar and radiating septa was composed of hyalinised collagen (Fig. 3a). Immunohistochemical analysis (IHC) revealed for the cystic lesion negativity for chromogranin A, synaptophysin and vimentin (Fig. 3b–d).

## Discussion and conclusions

SCAs are rare, benign neoplasms of pancreas [1]. The mean age of presentation is 60 years and women are more frequently affected. The majority of SCAs are asymptomatic and incidentally discovered. Abdominal pain, nausea and palpable mass are the most common presenting symptoms [7]. International guidelines stated that SCA is a benign entity [8]. For this reason, surgical treatment of these lesions is not recommended, except in case of patients with symptoms related to the compression of adjacent organs (bile duct, stomach, duodenum, portal vein) [8]. However, in literature large series of resected pancreatic cystic lesions comprise patients with a final diagnosis of SCA, including 40–60% of asymptomatic SCA [2, 9, 10]. The reason of this not negligible rate of “useless” resection for asymptomatic SCA is that in some cases can be difficult to differentiate this cystic lesion from mucinous or neuroendocrine one [11, 12]. CT-scan, MRI and Endoscopic Ultrasound (EUS) are useful diagnostic tools, that should improve the accuracy of diagnosis, but they can’t delete the risk of misdiagnosis [13]. In fact, in the current case, the FNA procedure was not useful for diagnostic confirmation. The Fine Needle Biopsy (FNB) might be use as alternative investigation, in order to catch the core of pathology; nevertheless, FNB could be used also to perform cytological specimens (through tissue apposition).

One of the most difficult differential diagnoses of SCA is represented by neuroendocrine pancreatic neoplasms. Typically, neuroendocrine lesions, especially the well differentiated neuroendocrine tumors, appear as hyper-vascular masses on abdominal CT scan and 11% of them appear cystic. The peripheral rim hyper-enhancement is a typical feature described in 85% of cystic pancreatic neuroendocrine lesions, which is usually not present in SCA [11]. The 68Ga-DOTA_peptide PET-CT is now becoming the standard molecular imaging technique recommended in most current guidelines for diagnosis and staging of neuroendocrine tumours. There is a strong established application of the somatostatin receptor PET imaging in pancreatic neuroendocrine lesions, showing a sensitivity and predictive positive value up to 100% [2]; on the other hand, false positives have been reported [14]. This imaging finding mislead the diagnosis, suggesting a of neuroendocrine pancreatic neoplasm and implied a wrong indication to surgical treatment. The patient was asymptomatic/few symptomatic and he could have been theoretically managed with periodical follow-up. Mixed serous-neuroendocrine neoplasm (MSNN) of the pancreas is a distinct clinicopathologic entity, defined as a tumor containing two components with different pathologies [15]. Several cases of MSNN have been reported [16]. Our case was not a MSNN, because at pathological examination there wasn’t any evidence of neuroendocrine features. To our knowledge, in literature this case firstly reported a pancreatic SCA showing pathological tracer uptake at 68Ga-DOTA-peptide. For this reasons, we are not allowed to extend this evidence to all other pancreatic SCA. On the other hand, this finding has to be taken to into account, in order to minimize the risk of misdiagnosis in the differential diagnosis between SCA and neuroendocrine pancreatic lesions and to avoid an overtreatment of the patient. We strongly encourage future studies evaluating the relationship between pancreatic SCAs and Ga-60-DOTA-peptide.

## Data Availability

Yes.
